# 2251 Assessing research impact: It takes a team

**DOI:** 10.1017/cts.2018.276

**Published:** 2018-11-21

**Authors:** Ashley Dunn, Michelle B. Bass

**Affiliations:** Stanford University School of Medicine

## Abstract

OBJECTIVES/SPECIFIC AIMS: Dissemination of research findings through the published literature is a complex but critical part of the scholarly communication process. Additionally, this time point on the translational spectrum is a key objective of the National Clinical Association for Advancing Translational Sciences (NCATS). Tracking the dissemination of research outputs can be difficult to identify and evaluate. The purpose of this case study was 2-fold: (1) identify tools and resources available freely to the public and through university subscriptions used to assess research output; and (2) compare the effectiveness of these tools oat tracking output at different levels of granularity. METHODS/STUDY POPULATION: The authors, Spectrum staff (D.A.) and School of Medicine librarian (M.B.), attended webinars hosted by other Academic Medical Center libraries conducting work on impact tracking and learned from vendor product managers about available tools and resources during on-site campus visits. Publications from Stanford’s Clinical and Translational Science Award (CTSA) were used to track the diffusion of research outputs (e.g., number of citations, document types, research areas, relative citation ratio, CTSAs collaboration) via library subscription services (e.g., Web of Science and Scopus) and freely available tools (e.g., iCite and PubMed). RESULTS/ANTICIPATED RESULTS: The authors found certain tools were more inclusive in retrieving grant funded research outputs. For example, in the case of UL1 grant (UL1TR001085, UL1TR000093, UL1RR025744), on a grant-level output, there were discrepancies in the number of publications retrieved: (1) PubMed found 644 outputs; (2) Web of Science found 497 outputs; and (3) Scopus found 190 outputs. After de-duplication, the search across Web of Science (WoS), Scopus, and PubMed yielded 899 publications. In total, 389 outputs were unique to PubMed; 165 were unique to WoS; and 90 were unique to Scopus. Future analysis will be conducted to identify the source of unique outputs from each database (e.g., conference proceeding, specific journals). Additional analysis based on other units of research outputs (e.g., author-level outputs and article-level outputs) are expected to yield similar discrepancies. DISCUSSION/SIGNIFICANCE OF IMPACT: Citation analysis is a valuable method of assessing research output and, to a larger extent, research impact in a given field. It can help investigators illustrate qualifications for undertaking new projects, highlight collaborations across schools and departments, justify a grant renewal, and/or highlight accomplishments for promotion. However, systematic and comprehensive evaluations are needed in tandem with citation analysis/bibliometric analysis to assess the translation and uptake of research outputs and activities that result in research impact. Furthermore, both investigators and staff need adequate time and training to process research outputs/activities and to effectively organize them in easily understood visualizations.

